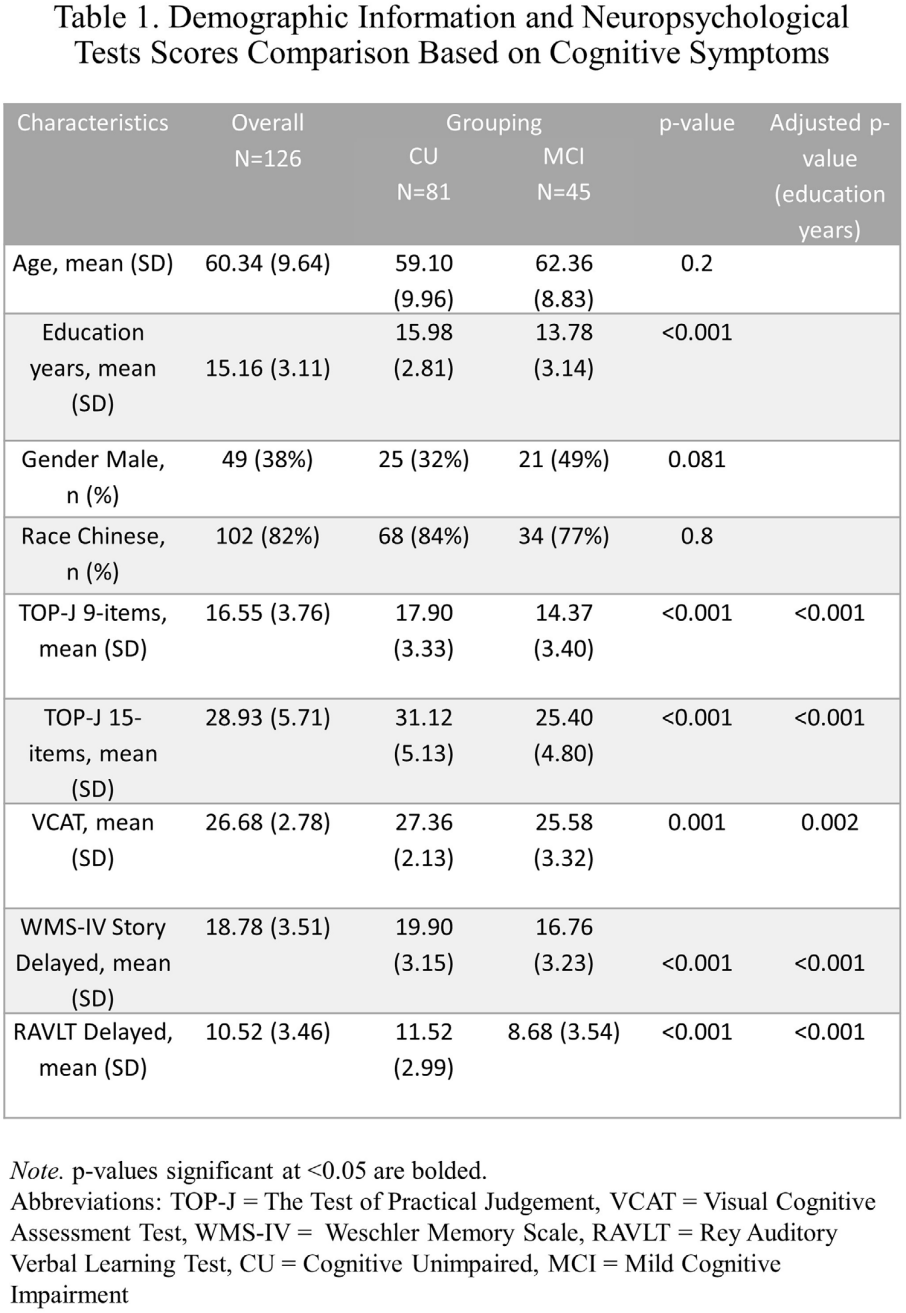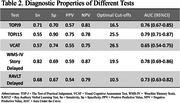# Usefulness of The Test of Practical Judgement for the detection of MCI in a Southeast Asian Population

**DOI:** 10.1002/alz.091207

**Published:** 2025-01-03

**Authors:** Pricilia Tanoto, Yi Jin Leow, Smriti Ghildiyal, Faith Phemie Hui En Lee, Shan Yao Liew, Isabelle Yu Zhen Tan, Wayne Freeman Chong, Eunice Limin Seah, Mohammed Adnan Azam, Ashwati Vipin, Gurveen Kaur Sandhu, Fatin Zahra Zailan, Farid Tan Bin Hasyim Tan, Nagaendran Kandiah

**Affiliations:** ^1^ Lee Kong Chian School of Medicine, Nanyang Technological University, Singapore Singapore

## Abstract

**Background:**

The Test of Practical Judgement (TOP‐J) in its 9‐item (TOPJ9) and 15‐item (TOPJ15) versions (Rabin et el., 2007), validated for assessing practical judgement, encompasses day‐to‐day scenarios in medical, financial, safety, and social/ethical domains. This study seeks to evaluate its utility in distinguishing cognitively unimpaired (CU) individuals from those with Mild Cognitive Impairment (MCI) within a Southeast Asian population. This investigation aims to contribute insights into the cross‐cultural applicability of TOP‐J in assessing practical judgement across varying cognitive states in the Southeast Asian context.

**Method:**

A cohort of 126 non‐demented participants from the Biomarker and Cognition Study, Singapore (BIOCIS) database underwent a comprehensive neuropsychological assessment, including TOPJ9, TOPJ15, Visual Cognitive Assessment Test (VCAT), Weschler Memory Scale (WMS‐IV) Logical Memory Story Delayed Recall, and Rey Auditory Verbal Learning Test (RAVLT) delayed test in a single session. MCI was diagnosed using the Petersen criteria (Petersen et al., 2001). Additionally, mean scores for neuropsychological tests were compared. Receiver Operating Characteristic (ROC) curve was used to assess the discriminative performance of TOP‐J between CU and MCI groups. Two‐by‐two tables facilitated the calculation of test sensitivity (Sn), specificity (Sp), positive predictive value (PPV), and negative predictive value (NPV) for each test.

**Result:**

The mean age of the 126 participants were 60.34 years ± 9.64, 38% male, mean education of 15.16 years ± 3.11, and 82% Chinese ethnicity. Significant mean differences between groups were observed for each test (p<0.05) (Table 1). ROC Analysis indicated optimal cut‐offs for MCI identification were determined for TOPJ9 (<16.5, AUC = 0.76, Sn = 0.71, Sp = 0.7, PPV = 0.57, NPV = 0.81) and TOPJ15 (<25.5, AUC = 0.79, Sn = 0.55, Sp = 0.9, PPV = 0.75, NPV = 0.78). Discriminative properties with story delayed and RAVLT delayed are detailed in Table 2.

**Conclusion:**

Both TOP‐J 9 and 15 items are useful for the detection of MCI in a Southeast Asian population. They exhibit comparable efficacy with other neuropsychological tests for MCI detection in Southeast Asian participants. Further validation through a longitudinal study is essential to solidify their diagnostic utility in this population.